# Qualitative research on end‐of‐life communication with family carers in nursing homes: A discussion of methodological issues and challenges

**DOI:** 10.1002/nop2.617

**Published:** 2020-09-19

**Authors:** Silvia Gonella, Paola Di Giulio, Alvisa Palese, Valerio Dimonte, Sara Campagna

**Affiliations:** ^1^ Department of Biomedicine and Prevention University of Roma Tor Vergata Roma Italy; ^2^ Azienda Ospedaliero Universitaria Città della Salute e della Scienza di Torino Torino Italy; ^3^ Department of Public Health and Pediatrics University of Torino Torino Italy; ^4^ Department of Medical Sciences University of Udine Udine Italy

**Keywords:** communication, end‐of‐life, family carers, nursing home, qualitative research

## Abstract

**Aim:**

To identify and summarize the challenges of conducting qualitative research exploring end‐of‐life communication between healthcare providers and bereaved family carers in nursing homes.

**Design:**

A descriptive qualitative study based on in‐the‐field‐notes and research diaries collected during a primary study involving 32 bereaved family carers and 14 nurses across 13 Italian nursing homes in 2018–2019.

**Methods:**

Two trained female nurses conducted semi‐structured, in‐depth, open‐ended interviews and recorded “in action” (i.e. reflections reported as in‐the‐field‐notes) and “on action” (i.e. retrospective reflections within the team reported immediately in the research diaries) narratives, with the aim of identifying challenges encountered during the research process. A content analysis process was performed to analyse the narratives collected.

**Results:**

We identified three major categories of challenges: (a) obtaining approval from the ethical committee; (b) approaching nursing homes and family carers; and (c) dealing with participant‐related impairments (i.e. memory, emotional, physical).

## 1 INTRODUCTION

Different types of end‐of‐life communication between healthcare providers (HCPs) and residents or their family carers occur in nursing homes (NHs), ranging from (a) “discussing” life‐sustaining treatments, care goals, advance directives, prognosis, the possibility of withdrawing treatments or palliative care options, (b) “speaking” symptom management and future care, (c) “talking” about how a patient is doing and (d) “receiving information” on a resident's health problems or what to expect (Gonella, Basso, Dimonte, et al., 2019).

Difficulties in end‐of‐life communication have been reported in NHs (Gjerberg, Lillemoen, Forde, & Pedersen, 2015) in addition to those documented in other settings (Prod'homme et al., 2018; Van Keer, Deschepper, Huyghens, & Bilsen, 2019; Xafis, Watkins, & Wilkinson, 2016). These usually entail late, infrequent, not intense or thorough enough or unclear communication with no or delayed sharing of care goals with residents and their relatives, often associated with an inadequate documentation of a resident's preferences.

Residents are infrequently involved in end‐of‐life communication since they are often cognitively impaired (Mitchell et al., 2009), or exploring their treatment preferences is postponed by HCPs due to emotional strain or lack of time until residents are no more cognitively competent (Bollig, Gjengedal, & Rosland, 2016). As a consequence, residents often receive aggressive treatments such as hospitalization, emergency room visits, parenteral or enteral nutrition (Mitchell et al., 2009; Basso, Simionato, Dimonte, Scaglione, & Campagna, 2018). Similarly, communication between HCPs and family carers is not always optimal nor timely (Caron, Griffith, & Arcand, 2005; Thoresen & Lillemoen, 2016). Around 75% of HCPs would talk to families about death, dying and treatment options only when the resident is approaching the end‐of‐life (Gjerberg et al., 2015), leaving them uninformed and unprepared for the upcoming death (Penders et al., 2015; Teno et al., 2011). However, an effective communication has been documented to elicit residents' preferences, thus promoting provision of care consistent with their wishes (Gonella, Campagna, Basso, De Marinis, & Di Giulio, 2019) and prepares relatives for death, offering emotional support and leading to informed decision‐making (Gonella, Basso, De Marinis, Campagna, & Di Giulio, 2019; Hebert, Schulz, Copeland, & Arnold, 2009).

Despite its relevance and the available qualitative (Bollig et al., 2016; Caron et al., 2005; Frey, Foster, Boyd, Robinson, & Gott, 2017) and quantitative studies (Mitchell et al., 2017; Reinhardt, Downes, Cimarolli, & Bomba, 2017; van Soest‐Poortvliet et al., 2014) on NH residents, relatives and HCPs communication, the challenges encountered during the research process as experienced by researchers have never been addressed to date. Discussing and reporting issues in designing and implementing study protocols about sensitive topics can increase a researcher's awareness, as well as can suggest how to design further studies to overcome challenges and to promote ethically and methodologically sound studies.

## 2 BACKGROUND

In the last two decades, qualitative studies have become one of the main research methods used by the caring sciences, especially in nursing and socially oriented disciplines (Cleary, Horsfall, & Hayter, 2014b). Due to their capacity to explore human and social experiences, as well as expectations, meanings and processes including those concerning end‐of‐life communication, qualitative research methods might help to identify the in‐depth implications of professional, organizational and/or policy interventions (Greenhalgh et al., 2016; Malterud, 2001). Qualitative methods have been identified as powerful tools to assess indirectly the quality of care by exploring patient's satisfaction (Fawsitt et al., 2017; Palese et al., 2017) and thus further informing policy‐decision makers (Bomhoff & Friele, 2017).

Conducting qualitative research is not as easy as it may appear; alongside, the challenges of identifying appropriate research question(s), methodology(ies) and study design(s) (Khankeh, Ranjbar, Khorasani‐Zavareh, Zargham‐Boroujeni, & Johansson, 2015), there are major challenges in study protocol implementation, for example when interviewing patients with Huntington's or Parkinson's disease that may suffer from speech or cognitive impairments (Cleary et al., 2014b; Khankeh et al., 2015; LaDonna & Ravenek, 2014).

Some challenges can be seen as unanticipated difficulties (Bail et al., 2019) and thus worthy of being reported and discussed, given their value in informing future researchers expected to design appropriate strategies. Other challenges can inform on the feasibility and the acceptability of certain research processes in a specific field. Feasibility involves an assessment to determine whether a research protocol is likely to be successfully implemented (Mesly, 2017). On the other hand, acceptability refers to the extent to which the protocol is considered appropriate (Vlassenroot, Brookhuis, Marchau, & Witlox, 2008).

Thinking critically about challenges encountered during a research process and being transparent about these challenges is an ethical imperative for researchers to conduct methodologically rigorous qualitative studies and improve available knowledge on methods by including their frameworks insights and evidence as emerged from their practical implementation (Ravenek & Rudman, 2013; Tracy, 2010).

The aim of this study was to identify and summarize the challenges encountered in designing and conducting qualitative research when exploring end‐of‐life communication between HCPs and bereaved family carers in Italian NHs. Our research question was as follows: Which are the challenges that researchers have to face when designing and conducting qualitative research about end‐of‐life communication between HCPs and bereaved family carers in NHs?

## 3 METHODS

### 3.1 Study design

A concurrent descriptive qualitative study design (Sandelowski & Barroso, 2006) was used while implementing a primary study on NH bereaved family carers. Methods have been reported here according to the COnsolidated criteria for REporting Qualitative studies (COREQ) guidelines (Tong, Sainsbury, & Craig, 2007).

### 3.2 Primary study

A qualitative descriptive study (Gonella, Basso, Clari, & Di Giulio, 2020; Gonella, Basso, Clari, Dimonte, & Di Giulio, 2020) involving 32 family carers and 14 nurses from 13 Italian NHs was conducted from December 2018–May 2019 with the primary intent of assessing the extent to which bereaved family carers of NH residents felt involved in end‐of‐life communication.

Fifty‐two NHs were approached and 13 joined the study on a voluntary basis. Family carers were eligible if (a) they were willing to participate, (b) their relative had spent the last 30 days of their life in the NH and (c) had died between 45 days–9 months prior to the study start. Family carers were identified by the NH director with the support of HCP(s) and then contacted according to NH preferences: (1) by phone call after preliminary contact by the NH director who provided an explanation of the study aims and requested permission for contact; (2) by phone call and interview directly scheduled by the NH director; or (3) with a personalized letter of condolences with a brief presentation of the study aims and the phone number of researchers to be contacted if interested in the study.

The nurse most often involved in the care during the resident's last week of life was also interviewed to explore his/her perceptions regarding end‐of‐life communication with family carers.

### 3.3 Data collection process

Two trained female nurses who were not affiliated with the NHs' participants or staff or to the family carers were responsible for data collection. Specifically, they were (a) a doctoral candidate in nursing with postgraduate specialization in bioethics and experienced in qualitative research and (b) a nurse with experience in NH end‐of‐life care, respectively. Semi‐structured, in‐depth, open‐ended interviews with follow‐up questions at the family carer's preferred location were carried out. Nurses mainly involved in the care of the residents were interviewed at their workplace before, at the end or during their shift when possible.

Data were collected through the in‐the‐field‐notes and a research diary. In in‐the‐field‐notes, defined as written narratives of observational data emerged by fieldwork including descriptive and interpretive data based on the observational experience of the researcher (Jackson, 1990), researchers were asked to report their “in action” reflections (Janssens, Bos, Rosmalen, Wichers, & Riese, 2018; Schon, 1984). In the research diary, as the repository for personal reflections and here used as a data collection tool (Snowden, 2015), the researchers were asked to report their “on action” reflections (Janssens et al., 2018; Schon, 1984).

Specifically:

(a) the “in action” or the “here‐and‐now” reflections were collected at the beginning of the research process, such as the ethical committee approval and the request for participant consent; and during the research process, with regard to: (i) the interview plan (whom to interview; how to reach them; how to approach them; how to word, order and pose questions; how much personal information, if any, to share; whether to play the role of a naive or informed listener; how to record what was being said—tape or notes; and when to stop) (Pawluch, 2005); (ii) the setting for the interview (i.e. NH, interviewee's house or coffee shop); and (iii) the appearance and demeanour of participants (emotional status—i.e. smile, crying; and non‐verbal behaviours—i.e. handwringing, lack of eye contact).
Brief, keyword‐based notes were taken during the interview, while maintaining eye contact with participants. The detailed field notes were dictated immediately after the interview ended, thus ensuring the researcher's memory was fresh, the prevention of recall bias and allowing for a free‐flow of ideas.
(b) the “on action” reflections were the in‐depth, retrospective reflections collected after each of four formal meetings organized over the study process in research diaries (Berger, 2015). These meetings were performed (i) after having obtained the approval from the ethical committee board; (ii) after having recruited NHs and family carers; (iii) immediately after each interview: the researcher phoned another member of the research team to share perceptions about the performance as an interviewer, the interviewer's and interviewee's feelings and how to overcome challenges encountered; and (iv) after the transcription of all the in‐the‐field‐notes.

Narratives, which emerged whatever challenges in (1) designing or (2) conducting qualitative research exploring end‐of‐life communication in NH, were all included. Practical non‐methodological challenges (i.e. reaching NH or family carers at long distances) were excluded.

### 3.4 Ethical considerations

The Ethics Committee of the University of Turin (Italy) approved the primary study (Reference 457626/10.12.2018). In the primary study, all participants gave their written informed consent to participate in the study and to be audio‐recorded after being informed about the study purpose and the process of data collection. Participants were free to participate and could stop the interview at any time and for any reason, without the need to give explanations. Transcriptions were anonymized with regard to both the names of the participants and the NHs. Moreover, the two researchers, who collected the data of the present study, gave their informed consent for the data collection process.

### 3.5 Data analysis

Direct content analysis (Graneheim & Lundman, 2004) was performed according to the following steps: (1) familiarization: all narratives reported in the in‐the‐field‐notes and in the research diaries (hereafter, narratives) were read carefully and repeatedly, first longitudinally (each interview, all narratives) and then horizontally (all interviews, by reading the narratives for the same question of the interview guide across all the interview transcripts); (2) compilation: two researchers (SG, PDG) independently examined each narrative line‐by‐line using an open coding approach whereby the most significant words and phrases (*units of meaning*) were highlighted; (3) condensing: two researchers (SG, PDG) independently reduced each meaningful unit to a descriptive label (*code(s)*); and (4) categorization: all researchers compared the codes and grouped them into *sub‐categories* according to their similarities; the sub‐categories were finally abstracted in *categories*. The analytical process is reported in Table 1. Any disagreements were solved by discussion within the research group. An audit trail was kept across the data analysis process; specifically, an example of the process is detailed in Table 2.

**TABLE 1:** Analytical process performed: steps followed

1. SG and PDG independently read the narratives and familiarized with the data
2. SG and PDG independently identified units of meaning and preliminary codes
3. SG, PDG, AP, VD and SC discussed and compared the preliminary codes until agreement was achieved
4. SG, PDG, AP, VD and SC grouped codes into sub‐categories and then categories; they agreed on the final codes and categories of methodological challenges of conducting qualitative research exploring end‐of‐life communication
5. SG checked the narratives to question the identified categories of methodological challenges and selected illustrative quotes that proved the findings
6. SG, PDG, AP, VD and SC discussed the identified categories of methodological challenges and illustrative quotes, and agreed about the interpretation of the data

Note

SG, PDG, AP, VD, SC, see authors.

**TABLE 2:** Analytical process performed: an example

Units of meaning	Codes	Sub‐categories	Categories
A NH director said: “Family carers are constantly informed about their relative's care. I do not feel the further need to go through these discussions about death and dying”	NHs' fear to be negatively judged for their working process	How to reach the potential participants	Approaching nursing homes and family carers
A NH director said: “This is an extremely interesting project but I do not know if we can share this data”	NHs' fear to violate residents' privacy		
A NH director said “I'll phone Ms X and schedule the interview if she desires to participate to this study; then I'll let you know day and time”	Interview scheduled by the NH director	How to approach potential participants	
A NH director said “I'll explain Mr X the study aims and request his permission for contact. Then, I'll give you his phone contact if he authorizes me”	Family carers approached by phone call		
A NH director said: “I think that family carers should be approached by letter; you can report your phone number that family carers interested in the study can call”	Family carers approached by letter		
A researcher said: “The madam was died almost 8 months before the interview but her daughter remembered all the details of her mother's last week of life”	Ensure intact memories with a long time frame	When to approach participants	
A researcher said: “The death was recent, only two months ago, but the daughter had difficulties in remembering several details of end‐of‐life communication, she had reset to forget the suffering of her mother's last days”	Prevent recall bias with a strict time frame		
A researcher said: “The saturation concept is not adequate for qualitative descriptive study design as ours”	Avoid the saturation concept	How many participants to enrol	
A researcher said: “We should avoid the principle thereby the sample size in a qualitative study should be sufficiently large and varied to elucidate the aims of the study since it does not provide guidance for planning”	Avoid the common principles to determine sample size		
A researcher said: “We need a tool based on shared methodological principles for estimating an adequate number of participants, such as the pragmatic model of ‘information power’”	Apply information power model		

Abbreviation: NH, nursing home.

### 3.6 Trustworthiness

A team member (AP) expert in qualitative research assessed compliance with guidelines for trustworthiness (Lincoln, 1995). To ensure credibility and dependability (Polit & Beck, 2008), when it was not possible to collect in‐the‐field‐notes (e.g. telephone interview), additional relevance was given to the interviewees' feedback on the transcribed interview. Repeated reading of the narratives and repeated discussions of the emerging categories which illustrated the methodological challenges of conducting qualitative research exploring end‐of‐life communication in addition to repeated discussions about alternative interpretations of the findings within the research group were performed to promote reflection (Darawsheh, 2014) and to validate the findings. Discussion on the identified categories of methodological challenges and illustrative narratives on completion was also performed to ensure confirmability (Polit & Beck, 2008).

## 4 RESULTS

**FIGURE 1:**
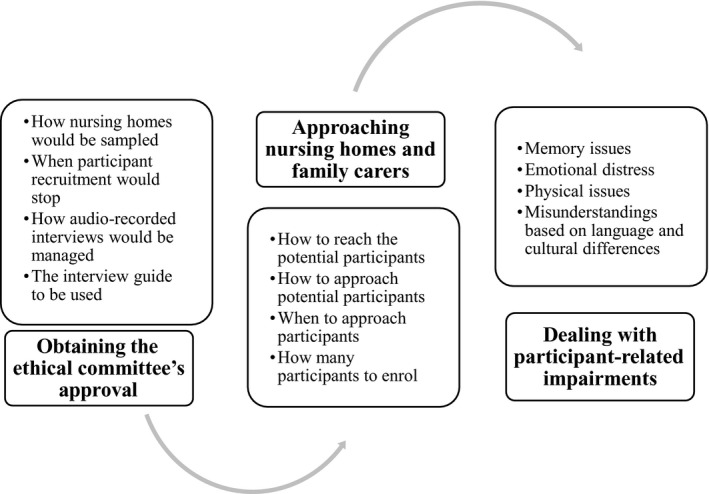
Major challenges while designing or conducting qualitative research exploring end‐of‐life communication in nursing home

We identified three major categories of challenges while designing and implementing the primary qualitative study on NH bereaved family carers: (1) obtaining the ethical committee's approval; (2) approaching NHs and family carers; and (3) dealing with participant‐related impairments (Figure 1).

### 4.1 Obtaining the ethical committee's approval

The study protocol was submitted to the Ethical Committee in June 2018 and approved in December 2018. Since all the Ethical Committee forms were designed for trials and quantitative studies, it was extremely challenging to fit the contents of our qualitative study. Phone calls were necessary to explain the nature of qualitative studies and the impossibility of completing all the fields required. We were finally required to provide further information about: (a) how NHs would be sampled; (b) when participant recruitment would stop; (c) how audio‐recorded interviews would be managed; and (d) the interview guide to be used.

We thus amended the protocol by better specifying, for each point arose above‐mentioned, the following revisions:

- “Participation to the study is not mandatory and NHs will adhere on a voluntary basis.”
- “Statistical sample estimations for generalization is inappropriate in qualitative research. Data saturation (i.e. redundant signs inform researchers that data collection may cease) and information power (i.e. the number of participants depends on the amount of information the sample holds) better fit the aim of qualitative research.”
- “Audio recordings will be stored in the data folder of an academic computer that will be accessible only to the research team.”
- “It is not possible to provide the definitive interview guide, that can be modified as new insight unfolds with interviews. The concept of “open protocol” is an essential part of qualitative research.”

### 4.2 Approaching NHs and family carers

#### 4.2.1 How to reach the potential participants

Several of the NHs initially approached seemed suspicious of the research project and were not interested in being part of the study. NHs might perceive themselves as being judged for their work on symptom control, treatment strategies and communication with families:

A NH director said: ‘Family carers are constantly informed about their relative's care, I do not feel the further need to go through these discussions about death and dying’.

Participation was further discouraged by the recently modified European regulation on data protection rules (Regulation (EU) 2016/679, 2016) which increased NH directors' worries of violating residents' privacy. Only a few NHs initially agreed to take part in our project. We then completed the recruitment of NHs by adopting a “snowball” strategy: participating NHs were asked to suggest other NHs that would potentially be interested in this project, with a final sample that was likely to be over‐representative of the palliative care culture.

#### 4.2.2 How to approach potential participants

In our study, family carers were approached after their relative's death. To guarantee sensitive data protection, to be as non‐intrusive as possible and to limit possible refusals for study participation, the NH director made first contact with the potential participants to inform them about the study, to verify their willingness to participate and to request permission for contact from the researchers. We therefore tailored the contact with families according to NH preferences, introducing a great diversity in the approaches used. Twenty‐two family carers joined the study after the researcher phone call; six interviews were directly scheduled by the NH director and only four family carers answered the personalized letters of condolence. Twenty family carers declined to participate as they were not interested in the project aims or remembering their relative's death was too challenging:

A researcher recorded that some family carers said ‘We witnessed uncontrolled suffering during the last days of life’, or ‘We felt somehow guilty for not having done as much as possible’.

Thus, we mainly approached participants who had mainly positive end‐of‐life experiences, while those with particularly distressing and burdensome end‐of‐life experiences who may have benefited from sharing their experience with researchers to receive emotional support, were less approached.

#### 4.2.3 When to approach participants

We set our time frame as between 45–90 days after the resident's death, to avoid approaching families during the acute bereavement stage. Due to difficulties in accessing participants in this timeframe, we also included carers whose relative had died up to nine months earlier. A stricter time frame may not prevent recall bias: some carers whose relative had died less than 2 months previously had forgotten several details about end‐of‐life communication and others who had lost their beloved long time ago had intact memories:

The death was recent, only two months ago, but the daughter had difficulties in remembering several details of end‐of‐life communication, she had reset to forget the suffering of her mother's last days.

#### 4.2.4 How many participants to enrol

The notes emerged during the study process about the decision on how many participants to involve led to embrace an innovative and emerging approach in the primary study named “information power” (Malterud, Siersma, & Guassora, 2015). According to this strategy, the more information the sample holds, the fewer participants are needed and this depends on the study aim (i.e. narrow versus broad), on the sample specificity (i.e. high versus sparse), on the use of established theory (i.e. level of theoretical background), on the quality of the dialogue (i.e. clear and focused interview versus ambiguous and unfocused interview) and on the strategy of analysis (in‐depth analysis of narratives versus exploratory analysis) (Malterud et al., 2015). Although our study aim was narrow and supported by a large amount of literature on end‐of‐life communication in NHs, the interview guide of the primary study was focused on the research question and the sample was highly specific, the choice to adopt an exploratory analysis strategy to investigate the broadest possible range of end‐of‐life communication dynamics increased the number of participants needed to offer sufficient information. We finally decided to recruit at least 30 participants although 12 interviews were suggested as sufficient to understand the common perceptions and experiences among a group of relatively homogeneous individuals (Guest, Bunce, & Johnson, 2006), according to the key principle of qualitative research of including a limited number of subjects who should be studied intensively (Cleary, Horsfall, & Hayter, 2014a).

### 4.3 Dealing with participant‐related impairments

Participants reported a variety of issues that may have negatively influenced the data collection process. Some of the difficulties encountered were embedded in the qualitative research model while others were specific to the population under study, namely relatives of NH residents:

(a) *memory issues*: some participants suffered from memory problems and/or had slowed thinking. Participants were allowed, if they wished, to read the topic guide, which was sent by email, WhatsApp or given in person before starting the interview, according to their preferences. Appropriate time was always allowed for answering; questions were rephrased and continuously recalled to support participants in answering; some questions were also modified to a closed version (yes/no):

The interviewer had to rephrase the question about satisfaction with end‐of‐life care three times as the daughter didn't understand.

(b) *emotional distress*: some participants had very challenging end‐of‐life experiences and felt the need to share many details not always consistent with our research aims. For some of them, the interview was a relief valve allowing them to talk about their experiences in the NH, for others it was a way to remember their dead relatives:

The daughter thanked me at the end of the interview since she had the opportunity to talk about her mother after many months.

It was sometimes necessary to allow them to go off‐topic or to be interviewed together with another bereaved family member to help them face such an emotionally challenging dialogue. Three interviews were therefore conducted with more than one person at once (two interviews with child and daughter‐in‐law and one interview with two nieces):

The niece asked to be interviewed together with another cousin who had been highly involved in the aunt's care at the end‐of‐life since those memories were too agonizing.

The location of the interview was decided by the family carer to allow maximum comfort. The interview with the nurse most involved in the resident's care during their last week of life was scheduled before that with the family carer, so as to gain a clinical view of the case and avoid upsetting errors with family carers;

(c) *physical issues*: some participants were of advanced age and were unable to drive or being transported; others chose to be interviewed in the NH facility so as to meet the NH personnel and/or residents with whom they had established a friendly relationship during the NH stay:

A 70‐years old son happily adhered to the project but asked to be interviewed at home since he hadn't any car neither could be transported to the NH by anyone.

Two participants were interviewed over the telephone since they lived abroad and this had not been provided for in the study protocol;

(d) *misunderstandings based on language and cultural differences*: in talking with the family carers, it was important to share the same dialect and language, giving a sense of closeness; and also, some interviewed nurses were non‐Italian workers with poor language proficiency:

A nurse perceived to be subordinate to the physician and tried to avoid answering critical questions by saying ‘Ask the physician, he will answer better than me’.

## 5 DISCUSSION

Our study was designed to identify challenges in undertaking qualitative research in end‐of‐life NH family carers communication. We identified three major challenges, one in the first stage during project approval and the others during the data collection process. Discussing these challenges is an ethical imperative so as to develop the researchers' ethical sensitivity and improve the rigour of qualitative methods to develop evidence capable of improving clinical practice. If these challenges are not addressed and remain unresolved, research findings may not be fully useful (i.e. the knowledge emerged is not‐ practical and practicable), appropriate (i.e. it does not fit a situation) or meaningful (Hannes & Harden, 2011).

We encountered difficulties in obtaining ethical approval mainly due to formal aspects of the study, rather than to the content of the research project itself. Difficulties in obtaining ethical approval for multicentre research have already been reported (Nicholl, 2000), suggesting that Ethics Committees should be more aware of qualitative research designs and arrange for specific documentation and appropriate criteria for analysing qualitative research protocols, considering issues such as criteria to stop information gathering, methods of data analysis to improve validity (i.e. triangulation) and that data collection methods may develop as the study unfolds (Peter, 2015). Ethics Committees should also develop critical thinking skills to distinguish good from poor qualitative research, thus identifying qualitative protocols that can offer promising results to inform clinical practice (Iannamorelli & Tognoni, 2017).

The sensitive topic we aimed to explore made NHs' recruitment challenging due to the local culture of death and palliative care, also influenced by the NH managers and the staff's leadership and cultural background (Bruera, 2004; Rivolta, Rivolta, Garrino, & Di Giulio, 2014). In Latin countries (i.e. Italy where the research took place), death and dying are still considered a taboo and HCPs often prefer to adopt behaviours of avoidance and concealment (Rivolta et al., 2014). End‐of‐life communications about poor prognosis are often scant due to an ingrained Catholic ethics aimed at maintaining hope (Toscani & Farsides, 2006). Some NHs may have been disinclined to lay bare problems in the communication with relatives and fear that their work will be negatively judged may have led them to refuse participation. It is likely that only the NHs more sensitive to palliative care and that wanted to call into question and improve the quality of their care joined the study (Norton, Ladwig, Caprio, Quill, & Temkin‐Greener, 2018). In this respect, the transparency of study aims and a non‐judgemental attitude are of utmost importance.

Self‐selection of NHs and family carers, defined as the opportunity to exercise control over the decision to participate in the study or not (Berk, 1983), may have introduced a selection bias in our study. However, similar criticisms have been previously reported in the attempt to guarantee privacy and confidentiality (Bollig et al., 2016; Frey et al., 2017; Waldrop & Kusmaul, 2011). Moreover, no form of reward (i.e. money, gifts) was offered to help participation to avoid practical, methodological and ethical issues (Bruera, 2004; Head, 2009). Rewards can influence free participation and possibly lead interviewees to tell researchers what they wish to hear (Bentley & Thacker, 2004).

To avoid recall bias, the time frame from the resident's death should be long enough to reduce the burden on participants, but still enable them to recall details of their relative's final days (DiBiasio et al., 2015). Thus, we recruited family carers whose relative had died between 45 days–9 months prior, consistent with research which showed family carers usually being interviewed between 45 days (Vandervoort, Houttekier, Vander Stichele, van der Steen, & Van den Block, 2014)–23.8 months after their relative's death (Teno et al., 2011). However, we found instances of recall bias even with participants whose relative's death was close in time, most likely because forgetting emotionally challenging times worked as a protective mechanism. In contrast, others participants shared end‐of‐life experiences rich in details despite they had lost their relative for a long time. Previous research suggested that the size of the recall bias depends on characteristics of the individual and the value of the emotion recalled (Barrett, 1997).

Finally, several challenges were participant‐related (i.e. memory, emotional, physical issues or misunderstandings in language and culture) suggesting the need for tailored strategies. By allowing participants to read the topic guide, they were supported in dividing the entire experience into specific parts regarding relevant experienced situations concerning communication with the HCPs and involvement in decision‐making; moreover, expected questions reduced emotional distress. To avoid complicated language (Cleary et al., 2014b), repeated or rephrased unclear questions and simple yes/no questions were employed (Nygård, 2006). The latter were adopted if an interview became stuck to break through the situation and they were followed by further in‐depth probing questions.

To deal with emotional distress, as already noted, family carers were allowed to go off‐topic for a few minutes (LaDonna & Ravenek, 2014) or interviewers helped them to regain their balance if the interview became emotionally demanding, particularly if the death had not yet been completely elaborated. Moreover, interviews with two people at once were considered worthwhile, since they helped carers sustain the emotional burden and remember details together. Contrasting opinions may lead interviewees to fall into line with each other's perceptions, but this situation was unlikely in our study since the family carers involved in double interview generally had homogenous experiences.

If a face‐to‐face interview was not possible as participants lived abroad, they were interviewed by telephone. These issues were not considered in advance in the study protocol and hindered the recording of field notes that are recommended in qualitative research to ensure rich contextual information (Phillippi & Lauderdale, 2018).

Since misunderstandings based on language and cultural differences can affect the quality of the interview (Birks, Chapman, & Francis, 2007), both the interviewers in our study had background knowledge about end‐of‐life communication and were natives to the area, thus sharing a linguistic background, cultural beliefs and values with the participants. If interviewees hear local dialect or phrasing, they may feel more comfortable and relaxed, thus opening up to the interview and increasing their chemistry with the interviewer (Birks et al., 2007). If well‐conducted, such an interview can also promote a positive critical reflection on the lived experience (Gordon, 1998).

### 5.1 Limitations

Our study has several limitations. In first instance, we collected insights and reflections from two researchers with experience in this research field and this may have prevented further challenges from emerging. Secondly, narratives were collected via a specific research process and in a given cultural and social context and therefore the challenges that were identified cannot be generalized to other research fields (Cleary et al., 2014a; Malterud, 2001; Malterud et al., 2015).

Thirdly, although we reflected on the positionality of the researchers by reporting their training and experience, their prejudices (i.e. family carers may drop the relative off at the NH and not be interested in end‐of‐life communication) and emotional distress (i.e. eliciting family carers' suffering may make interviewers feel guilty (Mitchell, 2011; Nygård, 2006) and hastily end the interview) that may have affected the interpretation of the findings were not explored.

## 6 CONCLUSION

Conducting qualitative research with the family carers of deceased NH residents about their experience of end‐of‐life communication and involvement in care planning poses several unique challenges. Some challenges were unexpected (i.e. difficulties posed by the ethical committee), while others were expected (i.e. involving NHs and family carers) and suggest some important issues for feasibility and acceptability. Sharing the experience and pitfalls of undertaking qualitative research should be an ethical imperative, given that it is only through an in‐depth reflection of practical work that it is possible to identify difficulties and offer insights on overcoming problems, thus enhancing the methodological rigour of qualitative studies.

Ethical committees should be made aware of the importance and relevance of investigating lived experiences and perceptions as factors that may affect patient satisfaction and the course of illness. Changing the culture of individuals and institutions requires time, and however, more often asking ethical committees to arrange specific documentation for qualitative research protocols and providing NHs with opportunities to be involved in research projects can promote this change.

End‐of‐life research in NH with elder family carers requires researchers to develop specific skills to promote participation. Together with flexibility in arranging the location and timing of data collection, researchers should have empathetic and technical interview skills, including ethical responses to distress, strategies to avoid emotional involvement and possibly a common linguistic and cultural background, especially when culturally based topics are investigated, such as end‐of‐life communication. In Southern Europe qualitative research, courses are usually offered only at a high degree of education (i.e. Master of Science, PhD), whereas Northern Europe and North America show a different sensitivity, with most nursing Bachelor's programmes offering courses in qualitative methodologies. The greater competence of researchers from Northern Europe and North America in performing qualitative research is confirmed by the fact that most published qualitative research is being conducted in these countries. Southern Europe should develop a greater sensitivity towards this methodology by offering more educational opportunities to qualitative researchers to ensure rigorous qualitative studies on potentially emotionally challenging topics.

We have striven to be transparent in discussing the challenges we encountered while conducting qualitative research in NH about end‐of‐life communication. We hope that sharing the challenges and the strategies adopted to overcome them will be of help to other researchers. This should also be an encouragement to persist in investigating these sensitive issues and amplifying the voice of older individuals admitted to NHs, family carers, nurses and researchers, despite the difficulties that may be encountered.

## CONFLICT OF INTEREST

The authors have no conflicts of interest to disclose.

## AUTHOR CONTRIBUTIONS

SG, PDG and AP contributed to the study concept and design. SG and PDG collected data. All authors analysed the data and interpreted the results. SG and AP drafted the manuscript. All authors critically reviewed and revised the manuscript and approved the final version. All authors take responsibility for the integrity of the data and the accuracy of the data analysis.

## Data Availability

The data are not publicly available due to privacy and ethical restrictions.
